# The effect of DASH diet on glycemic response, meta-inflammation and serum LPS in obese patients with NAFLD: a double-blind controlled randomized clinical trial

**DOI:** 10.1186/s12986-023-00733-4

**Published:** 2023-02-14

**Authors:** Farnaz Rooholahzadegan, Sara Arefhosseini, Helda Tutunchi, Taghi Badali, Manuchehr Khoshbaten, Mehrangiz Ebrahimi-Mameghani

**Affiliations:** 1 Student Research Committee, Tabriz University of Medical Sciences, Tabriz, Iran; 2 Endocrine Research Center, Tabriz University of Medical Sciences, Tabriz, Iran; 3 Department of Internal Medicine, Faculty of Medicine, Tabriz University of Medical Sciences, Tabriz, Iran; 4 Nutrition Research Center, Department of Biochemistry and Diet Therapy, Faculty of Nutrition and Food Sciences, Tabriz University of Medical Sciences, Tabriz, Iran

**Keywords:** Dietary approaches to stop hypertension, Glycemic control, Inflammation, Lipoploysaccharides, Non-alcoholic fatty liver disease

## Abstract

**Background:**

As dietary approaches to stop hypertension (DASH) dietary pattern has been shown to be effective in hypertension and obesity, the present study investigated the effects of following DASH diet on glycemic, meta-inflammation, lipopolysaccharides (LPS) and liver function in obese patients with non-alcoholic fatty liver disease (NAFLD).

**Methods:**

In this double-blind controlled randomized clinical trial, 40 obese patients with NAFLD were randomly allocated into either “*DASH diet*” (n = 20) or calorie-restricted diet as* "Control”* (n = 20) group for 8 weeks. Anthropometric measures, blood pressure, glycemic response, liver enzymes, toll-like reseptor-4 (TLR-4) and monocyte chemoattractant protein (MCP-1) and LPS as well as Dixon's DASH diet index were assessed at baseline and after 8 weeks.

**Results:**

After 8 weeks, although all obesity indices decreased significantly in both groups, the reduction in all anthropometric measures were significantly greater in DASH *vs* control group, after adjusting for baseline values and weight change. Fasting glucose level decreased in both group, however, no inter-group significant difference was found at the end of study. Nevertheless, serum levels of hemoglobin A1c (HbA1c), TLR-4, MCP-1 and LPS as well as aspartate aminotransferase (AST) decreased significantly in DASH group, after adjusting for baseline values and weight change (*p* < 0.001, *p* = 0.004, *p* = 0.027, *p* = 0.011, and *p* = 0.008, respectively). The estimated number needed to treats* (*NNTs) for one and two grade reductions in NAFLD severity following DASH diet were 2.5 and 6.67, respectively.

**Conclusion:**

Adherence to DASH diet could significantly improve weight, glycemia, inflammation and liver function in obese patients with NAFLD.

## Introduction

Exposure to an “obesogenic” environment such as sustained positive energy balance due to increased food supply and the overconsumption of energy-dense low nutrient-dense foods as well as modern sedentary lifestyle leads to excessive intrahepatic fat accumulation and increased adiposity known as nonalcoholic fatty liver disease (NAFLD), as a public health issue [1–3]. NAFLD is considered as an umbrella term that includes different types of fatty liver diseases unrelated to alcohol consumption [4]. Due to the metabolic roots of NAFLD, it has been recently proposed that to rename NAFLD as metabolic dysfunction-associated fatty liver disease or 'MAFLD' [5]. A recent systematic review and meta-analysis through the extraction of available epidemiological data on fatty liver disease demonstrated that MAFLD has an astonishingly high prevalence rate in overweight and obese adults [6]**.** Moreover, the global prevalence of NAFLD among general population is projected to raise up to 33.5%, which is largely related to obesity epidemic [7]. Genetic and epigenetic factors such as insulin resistance (IR), inflammation, oxidative stress and changes in gut microbiota are involved in the pathophysiology of NAFLD known as "Multi-Hit" [8]. IR-disturbances in intra-cellular insulin signaling pathways- plays a fundamental role in NAFLD [9]. Furthermore, simple steatosis could be followed by a number of metabolic abnormalities such as decreased fatty acid oxidation in the liver, increased de novo lipogenesis and adipose tissue lypolisis resulted in IR and in turn, IR is associated with other endocrine and metabolic disorders [9, 10]. The inter-relationship between obesity and NAFLD has been frequently reported and indicating the role of adipose tissue in regulating endocrine signaling pathways such as hormones, adipokines and pro-inflammatory cytokine [11, 12]. NAFLD is also called as the hepatic manifestation of metabolic syndrome (Mets) because of the coexistence of visceral obesity, IR, dyslipidemia, and hypertension [8, 13]. In addition, hypertrophy/ hyperplasia of adipose tissue is considered as one of the contributing factor in a low-grade chronic inflammation and metabolic dysfunction known as “meta‐inflammation” [14]. It is caused by an increased macrophage accumulation and the release of adipokines, cytokines and chemokines e.g. tumor necrosis factor α (TNF-α), toll-like receptors-4 (TLR-4), monocyte chemoattractant protein (MCP‐1) and some interleukins (inc. IL‐6, IL‐8, IL‐1β) [14]. There is evidence that weight reduction can be effective in the secretion of inflammatory markers [14]. TLR-4 plays a vital role in recognizing lipopolysaccharides (LPS)- an indicator of pathogenic bacteria invasion- and mediates signaling to produce pro-inflammatory cytokines [14]. Moreover, excessive adipose tissues releases free fatty acids (FFAs) due to degradation of triglycerides [15]. LPS and FFAs bind to TLR4 of monocytes/macrophages and produce inflammatory mediators, and therefore, lead to the prolonged production of inflammatory cytokines [15].

Despite the lack of an approved therapeutic approach in the treatment of NAFLD, evidence-based guidelines establish the fundamental role of lifestyle modifications, particularly calorie restriction, healthy diet, and regular physical activity in improving hepatic steatosis and histological features of NAFLD [16, 17]. In clinical and preclinical studies, several natural compounds have shown favorable effects in the prevention, inhibition and treatment of metabolic disorders [18–23]. This evidence represents a promising strategy for NALFD, whose pathogenesis is multifactorial [24]. Patients with NAFLD mostly consume diet which is low in whole grains, cereals, fruits, and vegetables and high in red meat, refined-grains and sugars, typically named as Western dietary pattern [25–27]. Moreover, results of a meta-analysis revealed the role of red meat intake and soft drinks in increased likelihood of NAFLD [28, 29]. One of the dietary strategies that has been studied in NAFLD management is the Dietary Approaches to Stop Hypertension (DASH) dietary pattern approved in the prevention and treatment of hypertension [30]. Previous studies have also shown that DASH diet has beneficial effects on several other disorders including obesity, Mets, type 2 diabetes mellitus (T2DM), cardiovascular disease, and depression [31–33]. DASH diet underlines fruits, vegetables, low-fat dairy products, whole grains, poultry, fish, nuts, seeds, and legumes intakes accompanied by reduction in fats, red meat, sweets, and sugar-containing drinks [30]. Meanwhile, this diet is low in sodium (< 2400 mg/day) and saturated fat while rich in protein, fiber, calcium, magnesium, potassium, zinc, and folate [34, 35]. Watzinger et al. [36] in a cross-sectional study showed that the DASH score was correlated to lower liver fat content and NAFLD. Indeed, in a case–control study, an inverse association was found between adherence to a DASH-style diet and odds of NAFLD [37]. Similarly, Maskarinec et al. [38] in the Multiethnic Cohort Study showed that higher quality diets during mid-to-late adulthood were associated with a lower risk of NAFLD. Moreover, results of a recent nested case–control study in the Multiethnic Cohort revealed that higher DASH score was negatively correlated with NAFLD risk [39]. The only interventional study aimed to assess 8-week adherence to DASH diet compared with low calorie diet showed improvements in body weight, liver enzymes, IR, lipid profile, inflammatory and oxidative stress biomarkers [40]. As a systematic review and meta-analysis of randomized controlled clinical trials reported that DASH diet seems to be more suitable dietary approach for weight loss compared with low-energy diets and lack of interventional study in investigating the effect of DASH diet on NAFLD [41, 42], this study compared the effect of adherence to DASH diet compared with calorie-restricted diet (CRD) on glycemic response, meta-inflammation and serum LPS in obese patients with NAFLD.

## Materials and methods

### Study design

This double-blinded controlled randomized clinical trial was designed to examine the adherence to DASH diet compared with CRD on cardiometabolic, inflammatory biomarkers and LPS in obese patients with NAFLD. The study was conducted according to the Declaration of Helsinki and approved by the ethics committee of research vice-chancellor and also registered in the Iranian Registry of Clinical Trials (IRCT20100209003320N17). In addition, an informed consent form was read and signed by the patients at baseline.

### Participants

Sixty two males and females newly diagnosed patients with mild and moderate NAFLD aged 20–50 years with body mass index (BMI) = 30–40 kg/m^2^ were enrolled. NAFLD was confirmed by a single radiologist using ultrasonography (Sonoace X4 Medisio, South Korea) in a fasting state and then, liver steatosis severity was categorized into three grades, i.e. grade I as *"mild"*, grade II as *"moderate"* based on Hamaguchi et al. [43].

The exclusion criteria were as follow: alcohol consumption, pregnancy, breastfeeding, menopause, regular exercise, following weight loss diet 3 months before the study, taking medications such as anti-diabetic, anti-lipidemic, anti-hypertensive, antibiotics, corticosteroids, oral contraceptives and anti-inflammatory drugs, as well as suffering from liver, kidney, thyroid, gastro-intestinal, autoimmune diseases, T2DM, polycystic ovary syndrome (PCOS), and cancer.

### Sample size

According to mean and standard deviation of serum MCP-1 reported by Wamberg et al. [44] and by considering 95% confidence interval (CI) and %80 power among patients with NAFLD using sample size software (PASS; NCSS, LLC, US), sample size was found 20 for each group which then increased to 24 by considering 10% drop-out rate.

### Randomization, blinding, and intervention

To randomly allocate the patients into two groups, Random Allocation Software (RAS) and randomized block procedure were used. The patients were assigned into either DASH or CRD groups (1:1) by an assistant not involved in the trial. Size 3 randomized block procedure was applied as follows; gender (female *vs* male), age (18–35 yrs. *vs* 36–55 yrs.) and BMI (< 35 kg/m^2^ *vs* ≥ 35 kg/m^2^)]. Before randomization for treatment, the assignment was concealed.

Energy requirement was estimated individually according to Mifflin formula and weight loss diet was planned by reducing 500 kcal from the estimated energy for all the patients [45]. Macronutrient distribution was 55–60%, < 30%, and 10–15% of energy from carbohydrates, fat, and protein, respectively. Meal plans were prepared based on these calculations and the food-based dietary guidelines for Iranians (available at http://www.fao.org/nutrition/education/food-baseddietary-guidelines/regions/countries/iran/fr/) for CRD. For DASH diet, weight loss diet was designed according to DASH dietary pattern [46]. The DASH diet was rich in fruits, vegetables, whole grains, and low-fat dairy products and low in saturated fats, cholesterol, refined grains, and sweets. Suggested sodium in the DASH diet was < 2400 mg/day.

Food group exchange list and food album were delivered to each patient to follow the prescribed diet. The participants were also given a full explanation on how to use food exchange lists to replace the foods they did not have access to, by the foods of equal calorie from the corresponding food groups as well as delivering food group exchange list and food album to follow the prescribed diet.

### Assessment of anthropometric measures, dietary intake, and physical activity

At the beginning and end of the trial, personal and disease details, anthropometric measurements, physical activity levels and dietary intakes were assessed. Weight and height were measured using Seca stadiometer (Hamburg, Germany) to the nearest 100 g and 0.5 cm with low clothes without shoes, respectively. BMI was estimated as weight (Kg) divided by height squared (m^2^). The circumferences of waist and hip were also measured at the halfway between the lower ribs and the iliac crest and around the widest portion of the buttocks to the nearest 0.1 cm, respectively. Then, BMI, Waist-to-hip ratio (WHR) and waist-to-height ratio (WHtR) were estimated.

A 3-day food record was completed by the patients at baseline, week 4 and 8 and then, mean of each 3-day food record was calculated for each food item, converted to grams and ml, finally energy and macronutrient intakes were obtained using Nutrition IV software (First Databank: Hearst, San Bruno, CA, USA) at baseline and after 8 weeks.

Furthermore, Dixon’s DASH diet index was estimated to confirm adherence to DASH diet [46]. Foods were categorized into 9 components based on the following daily recommendations: total fruits (≥ 4 servings), total vegetables (≥ 4 servings), whole grains ((≥ 4 servings), low-fat dairy products (≥ 2 servings), legumes, seeds and nuts (≥ 4 servings), meat/meat equivalents (< 170 g), added sugar (< 3% of total daily energy), alcoholic beverages (≤ 2 drink) and saturated fat (< 5% of total daily). Foods were categorized into 9 components based on the following daily recommendations were scored 1 point and other than recommendation were scored zero point: total fruits (≥ 4 servings), total vegetables (≥ 4 servings), whole grains ((≥ 4 servings), low-fat dairy products (≥ 2 servings), legumes, seeds and nuts (≥ 4 servings), meat/meat equivalents (< 170 g), added sugar (< 3% of total daily energy), alcoholic beverages (≤ 2 drink) and saturated fat (< 5% of total daily). An overall adherence to DASH diet, therefore, was obtained by summing up the points ranging from 0 to 9 [47–49].

To assess physical activity level, the international physical activity questionnaire-short form (IPAQ-SF) was applied through face-to-face interview [50]. The patients were asked to report the time spent doing each of the defined intensity-varied activities during the past week to calculate metabolic equivalent of task (MET-hours/week) score. Then, the participants were categorized into: “low”, “moderate”, or “high” activity level [50].

### Laboratory assays

At baseline and at the end of study, after 12–14 h overnight fasting, blood sample was obtained from each patient and serum was separated. Metabolic factors including serum glucose, alanine aminotransferase (ALT) and aspartate transaminase (AST) concentrations were determined at the same day while the rest was stored at − 70 °C until assays. Serum alanine aminotransferase (ALT) and aspartate aminotransferase (AST) concentrations were assessed at baseline and at the end of study using the International Federation of Clinical Chemistry (IFCC) approved method. Hemoglobin A1C (HbA1c) was assessed using photometry in whole blood using Pars Azmoun Company kit (Pars Azmoun, Iran) and Hitachi auto analyzer (Hitachi-917, Tokyo, Japan). Furthermore, serum levels of TLR-4, MCP-1, and LPS were assessed using enzyme-linked immunosorbent assay (ELISA) kit (LSBio, Seattle, WA). According to complete blood count (CBC) results, white blood cell (WBC)-derived inflammatory indices including neutrophil to lymphocyte ratio (NLR), monocyte to lymphocyte ratio (MLR), platelets to to lymphocyte ratio (PLR), monocyte to high-density lipoprotein cholesterol (HDL-C) ratio (mHDL) and finally, systemic inflammation response index (SIRI)- as an index reflecting the host immune and inflammation balance—was estimated: SIRI = Neutrophil × monocyte/lymphocyte [51–53].

### Study outcomes

Changes in energy and macronutrient intakes, serum glycemic indices, MCP-1 and TLR-4, LPS, blood pressure, and anthropometric indices were considered as the primary outcomes whereas changes in serum levels of liver enzymes and NAFLD grade were considered as the secondary outcomes.

### Statistical analysis

All statistical analyses were performed using SPSS Statistics software (IBM SPSS Statistics, Armonk, USA, latest version). The distribution of continuous variables was checked using Kolmogorov–Smirnov test. For assessing both primary and secondary outcomes, after treatment approach was applied. Data were expressed as mean ± standard deviation (SD), median (min, max), and number (%) for continuous variables with symmetric and asymmetric distribution and categorical variables, respectively. Inter-group differences in the continuous and categorical variables at baseline were performed using independent samples *t*- and Chi-square tests, respectively. Paired samples *t*- and Sign tests were used for changes variables. At the end of the trial, the analysis of covariance (ANCOVA) test was used to compare between group changes in variables by adjusting for the confounders (i.e., baseline values and weight change). Absolute risk reduction (ARR) was calculated based on the difference in the event rate between DASH and control groups and then number needed to treat (NNT) was estimated according to the following formula: NNT = 1/ARR. The significance level was defined at p value lower than 0.05.

## Results

**Fig. 1:**
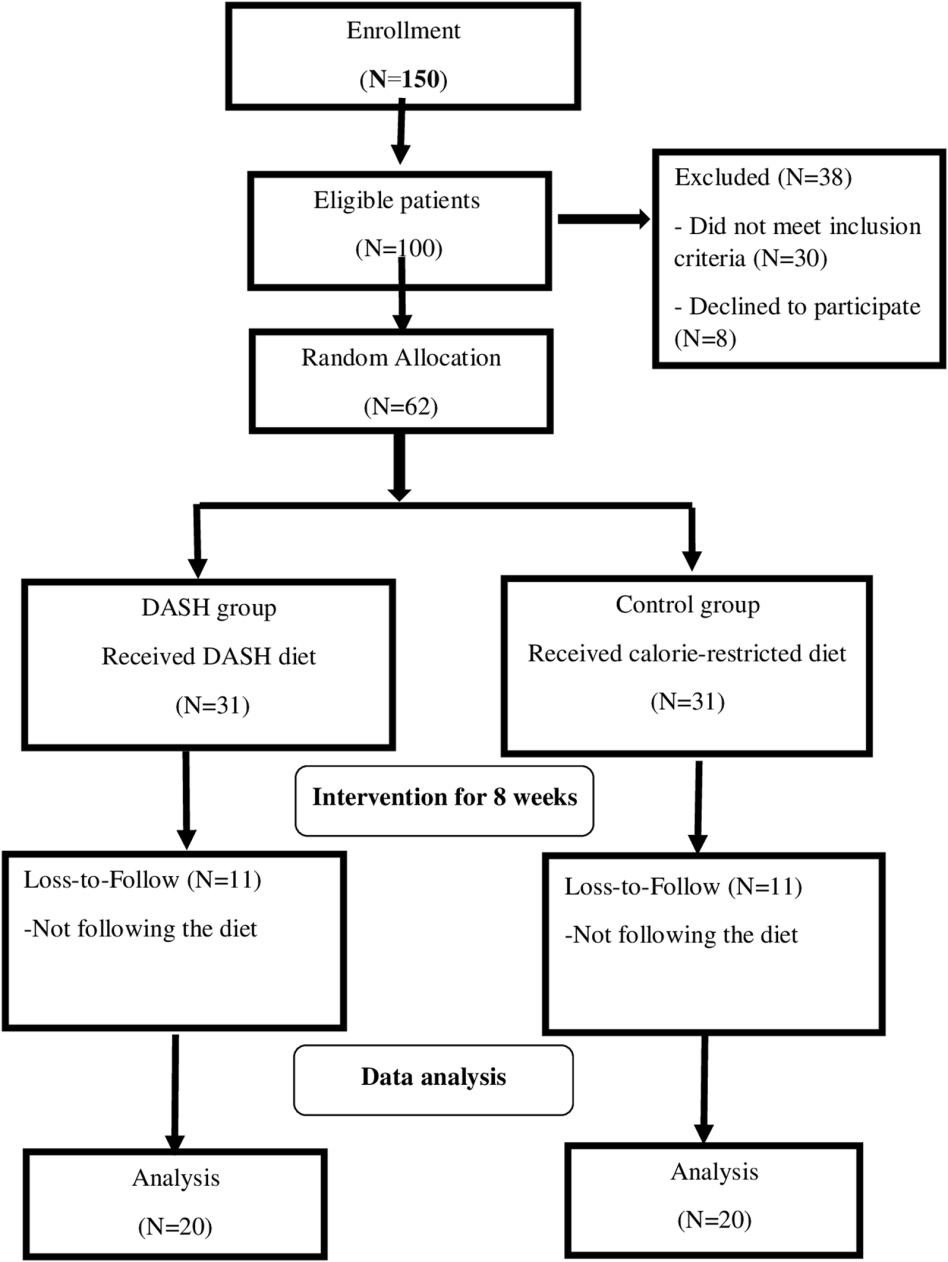
Flow chart of the study

Of totally 62 patients enrolled the trial, 40 subjects (20 patients in each group) completed the trial while 11 patients in each group lost to follow because of not following the prescribed diet (Fig. 1).

Table 1 demonstrates baseline characteristics in two studied groups. More than half of the studied patients in both groups were women and married. At baseline, no significant differences were found for not only demographic characteristics but also for NAFDL severity and physical activity level between the groups.

**Table 1:** Baseline characteristic of the study participants

Variable	DASH (N = 20)	Control (N = 20)	*p*
Age (yr.)	38.80 ± 9.98	37.10 ± 9.74	0.589*
Weight (Kg)	93.32 ± 19.51	93.49 ± 13.98	0.976*
Height (cm)	166.49 ± 12.0	165.52 ± 7.86	0.764*
BMI (Kg/m^2^)	33.43 ± 4.09	34.02 ± 3.61	0.632*

	N (%)	N (%)	
Female	13 (65.0)	12 (60.0)	0.594**
Married	17 (85.0)	15 (75.0)	0. 683**
*Educational level*			
Less than Diploma	6 (30.0)	3 (15.0)	0.520**
Diploma	6 (30.0)	9 (45.0)	
University degrees	8 (40.0)	8 (40.0)	
Physical activity level			
Light	10 (50.0)	11 (55.0)	0.674**
Moderate	7 (35.0)	8 (40.0)	
Heavy	3 (15.0)	1 (5.0)	
NAFLD severity			
Mild	10 (50.0)	12 (60.0)	0.751**
Moderate	10 (50.0)	8 (40.0)	

*DASH*, Dietary Approaches to Stop Hypertension, *BMI* Body mass index, *NAFLD* Non-alcoholic fatty liver disease. Data are presented as mean ± SD for numerical data and number (%) for categorical variables

*p value for Independent sample t-test

**p value for Chi square test

Changes in dietary energy and nutrient intakes over the study in control and DASH group are presented in Table 2. Apart from monounsaturated fatty acids (MUFA), dietary fiber, sodium and magnesium at baseline, no significant differences were found in not only energy and nutrient intakes but also the proportion of macronutrients from energy between the groups before and after the study. At baseline and end of the study, saturated fat intake in DASH group was significantly less than in control group (*p* < 0.001). Moreover, no significant changes were observed in physical activity level in both groups over the intervention (data not shown). Therefore, physical activity and dietary energy and nutrient intakes were not considered as confounders in data analysis.

**Table 2:** Daily dietary intakes before and after the study

Variable	DASH (N = 20)	Control (N = 20)	*P*
*Energy (Kcal)*			
Baseline	1585.60 ± 187.67	1629.50 ± 206.54	0.488**
End	1553.00 ± 134.07	1640.10 ± 174.38	0.104^***^
P*	0.326	0.895	
*Carbohydrates (g)*			
Baseline	227.55 ± 31.46	234.40 ± 36.20	0.527**
End P*	219.59 ± 22.94 0.243	232.80 ± 25.70 0.949	0.123***
*Protein (g)*			
Baseline	73.09 ± 8.17	75.72 ± 12.51	0.436**
End	69.52 ± 8.94	70.85 ± 13.68	0.978***
P*	0.192	0.164	
*Fat (g)*			
Baseline	46.17 ± 6.18	46.78 ± 10.11	0.841**
End	48.21 ± 5.61	49.84 ± 7.41	0.461***
P*	0.094	0.270	
*Carbohydrates (%)*			
Baseline	57.33 ± 2.28	57.07 ± 3.46	0.780**
End	56.54 ± 2.89	56.66 ± 3.60	0.910***
P^*^	0.290	0.692	
*Protein (%)*			
Baseline	18.47 ± 1.00	18.99 ± 2.26	0.354**
End	17.90 ± 1.66	17.46 ± 2.70	0.535^***^
P^*^	0.211	0.054	
*Fat (%)*			
Baseline	26.23 ± 2.03	25.96 ± 4.00	0.791**
End	27.94 ± 2.23	27.49 ± 3.13	0.609^***^
P^*^	**0.006**	0.109	
*SFA (g)*			
Baseline	9.37 ± 2.73	18.41 ± 5.66	< 0.001**
End	9.69 ± 2.00	17.39 ± 3.28	< 0.001^***^
P^*^	0.242	0.309	
*MUFA(g)*			
Baseline	21.09 ± 3.22	14.25 ± 3.99	< 0.001**
End	21.63 ± 3.45	15.60 ± 3.48	0.057^***^
P^*^	0.415	0.222	
*PUFA (g)*			
Baseline	8.89 ± 1.18	7.63 ± 4.45	0.227**
End	10.03 ± 1.93	9.82 ± 4.81	0.868***
P^*^	0.088	0.096	
*Dietary fiber (g)*			
Baseline	23.06 ± 6.45	17.89 ± 5.57	0.010**
End	21.93 ± 6.32	17.19 ± 6.32	0.794***
P^*^	0.065	0.346	
*Sodium (mg)*			
Baseline	925.69 ± 344.87	1341.12 ± 504.75	0.004**
End	987.39 ± 345.33	1277.44 ± 412.15	0.078***
P*	0.465	0.536	
Magnesium (mg)			
Baseline	277.76 ± 65.74	227.29 ± 69.42	0.023**
End	266.94 ± 70.42	211.73 ± 70.25	0.705***
P*	0.340	0.249	
*Potassium (mg)*			
Baseline	3181.75 ± 1457.01	2916.95 ± 834.20	
End	3510.35 ± 1016.14	2715.85 ± 970.19	0.485**
P*	0.106	0.296	0.530***
*Calcium (mg)*			
Baseline	856.98 ± 308.63	700.03 ± 269.69	0.095**
End	921.32 ± 291.93	592.40 ± 228.59	0.074***
P*	0.357	0.062	
*Vitamin D (µg)*			
Baseline	2.14 ± 1.21	1.81 ± 1.34	0.426**
End	2.25 ± 0.84	1.08 ± 1.11	0.091***
P^*^	0.850	**0.048**	

Bold indicates* p* < 0.05 is statistically significant

*DASH* Dietary Approaches to Stop Hypertension, *SFA* Saturated fatty acid, *MUFA* Monounsaturated fatty acid, *PUFA* Polyunsaturated fatty acid. Mean (SD) and Mean Difference (95% CI) are presented for data

*p value for paired- t test

**p value for Independent samples t-test

***p value for ANCOVA test (adjusted for baseline values and weight change)

Table 3 demonstrates changes in anthropometric measures, obesity indices, as well as metabolic and inflammatory biomarkers in studied groups. Apart from hip circumference** (**HC), the reduction in all anthropometric measures were significantly greater in DASH group than in control group, after adjusting for baseline values and weight change. Although fasting blood sugar (FBS) decreased in both groups, between-group comparison did not reveal any significant difference after 8 weeks, after adjusting for baseline values and weight change. Nevertheless, despite significant difference in serum HbA1c between the groups at baseline, a significant reduction in HbA1c was observed in DASH group (*P* < 0.001) while there was an increase in serum HbA1c in control group. Inter-group changes in serum HbA1c was statistically significant, after adjusting for baseline values and weight change (*p* < 0.001) (Table 3).

**Table 3:** Anthropometric measures, metabolic and inflammatory biomarkers before and after the study

Variable	DASH	Control	*P*
	(N = 20)	(N = 20)	
*Weight (Kg)*			
Baseline	93.32 ± 19.51	93.49 ± 13.98	0.976**
End	85.57 ± 18.62	87.88 ± 13.88	0.021***
MD (95% CI), P^*^	−7.75 (−9.34, −6.17), > **0.001**	− 5.61 (− 6.71, − 4.50). < **0.001**	
*BMI (Kg/m*^*2*^*)*			
Baseline	33.43 ± 4.09	34.02 ± 3.61	0.632**
End	30.64 ± 4.06	31.96 ± 3.57	0.025***
MD (95% CI), P^*^	−2.79 (−3.35, −2.22), < **0.001**	−2.06 (−2.47, −1.64), < **0.001**	
*WC (cm)*			
Baseline	111.25 ± 12.29	109.92 ± 9.80	0.708**
End	103.32 ± 12.67	105.0 ± 9.60	0.002***
MD (95% CI), P^*^	−7.92 (−9.58, −6.27), < **0.001**	−4.92 (−5.94, −3.91), < **0.001**	
HC (cm)	115.42 ± 10.47	115.92 ± 5.84	
Baseline	111.07 ± 11.44	112.20 ± 5.99	0.853**
End	−4.35 (−5.22, −3.48), < **0.001**	−3.72 (−4.63, −2.82), < **0.001**	0.323***
MD (95% CI), P^*^			
WHR		0.95 ± 0.07	
Baseline	0.96 ± 0.04	0.93 ± 0.07	0.406**
End	0.93 ± 0.05	−0.01 (−0.02, 0.00),** 0.031**	0.014***
MD (95% CI), P^*^	−0.03 (−0.05, −0.02), < **0.001**		
*WHtR*			
Baseline	0.67 ± 0.06	0.66 ± 0.05	0.821**
End	0.62 ± 0.06	0.63 ± 0.05	0.002***
MD (95% CI), P^*^	−0.05 (−0.06, −0.04), < **0.001**	−0.03 (−0.04, −0.02), < **0.001**	
*FBS (mg/dl)*			
Baseline	93.41 ± 9.63	93.12 ± 9.63	0.933**
End	90.76 ± 5.69	90.64 ± 4.69	0.923***
MD (95% CI), P^*^	−2.64 (−5.82, 0.53), 0.098	−0.21 (−0.37, −0.05), **0.013**	
*HbA1c (%)*			
Baseline	5.30 ± 0.35	5.49 ± 0.49	< 0.001**
End	5.09 ± 0.34	5.56 ± 0.32	< 0.001***
MD (95% CI), P^*^	−2.49 (−7.54, 2.57), 0.314	0.07 (−0.08, 0.21), 0.340	
*TLR-4 (ng/ml)*			
Baseline	0.82 ± 0.11	0.80 ± 0.17	0.656**
End	0.70 ± 0.13	0.82 ± 0.14	0.004***
MD (95% CI), P^*^	−0.13 (−0.20, −0.05),**0.003**	0.02 (−0.05, 0.08), 0.634	
*MCP-1 (pg/ml)*			
Baseline	110.75 ± 7.56	11.64 ± 16.48	0.828**
End	100.44 ± 10.69	110.01 ± 11.79	0.027***
MD (95% CI), P^*^	−10.32 (−16.36, −4.27), **0.002**	−1.64 (−8.12, 4.85), 0.603	
*NLR*			
Baseline	1.92 ± 0.81	1.81 ± 0.64	0.238**
End	1.94 ± 0.83	1.71 ± 0.60	0.176***
MD (95% CI), P^*^	26.51 (19.08, 33.94), < **0.001**	37.44 (29.64, 45.23), < **0.001**	
*MLR*			
Baseline	0.17 ± 0.09	0.16 ± 0.07	0.732**
End	0.23 ± 0.34	0.16 ± 0.08	0.179***
MD (95% CI), P^*^	0.06 (−0.09, 0.21), 0.399	0.004 (−0.16, 0.02), 0.701	
*PLR*			
Baseline	0.12 ± 0.03	0.11 ± 0.4	0.731**
End	0.12 ± 0.04	0.12 ± 0.05	0.019***
MD (95% CI), P^*^	−0.002 (−0.010, 0.006), 0.680	0.01 (0.001, 0.02),** 0.030**	
*SIRI*			
Baseline	688.53 ± 437.71	720.85 ± 622.22	0.850**
End	862.13 ± 1139.21	706.92 ± 610.50	0.151***
MD (95% CI), P^*^	173.59 (−308.57, 655.76), 0.460	−13.92 (−68.06, 40.22), 0.597	
*mHDL*			
Baseline	7.59 ± 3.67	10.56 ± 8.65	0.166**
End	13.47 ± 29.77	9.44 ± 6.70	0.080***
MD (95% CI), P^*^	5.87 (−7.30, 19.04), 0.362	− 1.12 (− 2.70, 0.45), 0.152	
*LPS (pg/ml)*			
Baseline	21.66 ± 1.83	20.72 ± 2.43	0.174**
End	18.91 ± 2.98	20.90 ± 2.36	0.011***
MD (95% CI), P^*^	−2.75 (−4.17, −1.33)**, < 0.001**	0.18 (− 1.18, 1.54), 0.785	
*AST (IU/L)*			
Baseline	24.10 ± 10.91	26.75 ± 9.28	0.413**
End	18.40 ± 6.57	25.05 ± 8.70	0.008***
MD (95% CI), P^*^	−5.70 (−9.42, −1.98), **0.005**	−1.70 (−3.53, 0.13), 0.067	
*ALT (IU/L)*			
Baseline	27.20 ± 14.0	37.35 ± 18.37	0.057**
End	18.75 ± 8.91	31.60 ± 16.24	0.149***
MD (95% CI), P^*^	−8.45 (−12.89, −4.01), **0.001**	−5.75 (−10.46, −1.04), **0.019**	

Bold indicates* p* < 0.05 is statistically significant

*DASH* Dietary Approaches to Stop Hypertension, *BMI* Body mass index, *WC* Waist circumference, *HC* Hip circumference, *WHR* Waist to hip ratio, *WHtR* Waist to height ratio, *FBS* Fasting blood sugar, *HbA1c* hemoglobin A1c, *NLR* Neutrophil to lymphocyte, *MLR* Monocyte to lymphocyte, *PLR* Platelets to lymphocyte, *SIRI* Systemic inflammation response index, *mHDL* Monocyte to high-density lipoprotein cholesterol ratio, *AST* Aspartate aminotransferase, *ALT* Alanine aminotransferase, *TLR-4* Toll-like receptor-4, *MCP-1* Monocyte chemoattractant protein-1, *LPS* Lipopolysaccharides

Mean (SD) and Mean Difference (95% CI) are presented for data

*p value for paired- t test;

**p value for Independent samples t-test;

***p value for ANCOVA test (adjusted for baseline values and weight change)

There were significant reductions in serum levels of TLR-4, MCP-1 and LPS in DASH group while no changes were found in these variables in control group (Table 3). After adjusting for baseline values and weight change, inter-group analysis showed significant differences in serum concentrations of TLR-4, MCP-1 and LPS. Among WBC-derived inflammatory indices, only PLR change was statistically significant between the two groups, after adjusting for the baseline values and weight change (*p* = 0.019).

Regarding serum liver enzymes, significant reductions in both serum levels of AST and ALT were found in DASH group whereas serum ALT decreased significantly in control group (Table 3). After adjusting for baseline values and weight change, there was only significant between-group difference in serum concentration of AST (*p* = 0.008).

Table 4 summarizes the effectiveness of DASH diet in NAFLD improvement. It was observed significant improvement in liver steatosis for both group, i.e. the greatest improvement as being free from NAFLD was seen for DASH group (80%) and control group (40%), respectively. The estimated NNTs due to 8-week following DASH diet compared with control group for one and two grade improvements in NAFLD severity were 2.5 and 6.67, respectively.

**Table 4:** Changes in liver steatosis

Variable	DASH (N = 20)	Control (N = 20)	P*
*NAFLD severity*			
Baseline			0.273
Grade I	10(50.0)	8 (40.0)	
Grade II	10 (50.0)	12 (60.0)	
*End*			
Grade 0	12 (60.0)	4(20.0)	**0.012**
Grade I	8 (40.0)	12 (60.0)	
Grade II	0 (0.0)	4 (20.0)	
*Liver steatosis severity*			
No change	1 (5.0)	12 (60.0)	
1 grade reduction	16 (80.0)	8(40.0)	
2 grade reduction	3 (15.0)	0 (0.0)	
*ARR*			
1 grade reduction (%)	40	–	
2 grade reduction (%)	15	–	
*NNT*			
1 grade reduction	2.5	–	
2 grade reduction	6.67	–	

Bold indicate* p* < 0.05 is statistically significant

^*^Chi square test

DASH, Dietary Approaches to Stop Hypertension; Non-alcoholic fatty liver disease; ARR, Attributable risk reduction; NNT, Number needed to treat

## Discussion

The results of the present study designed to examine the effect of adherence to DASH diet compared with CRD on glycemic response, meta-inflammation and serum LPS in obese patients with NAFLD showed greater reductions in weight and obesity indices, serum levels of HbA1c, AST, LPS and inflammatory biomarkers.

As energy for both DASH diet and CRD had been estimated based on Mifflin formula (around 1550–1650 kcal/day) with similar macronutrient distribution from energy, no significant differences in energy and macronutrient intakes were found between the groups (Table 2). Results of the estimation of Dixon's DASH diet index revealed good adherence to DASH diet i.e. score 8 to 9 (ranged 0–9) at baseline and end of the study, respectively. Therefore, changes in the study outcomes could be attributed to the weight loss intervention diet. Apart from saturated fat intake in DASH group which was approximately half of that in CRD group (*p* < 0.001) at baseline and end of the study, there were no significant differences in micronutrients intakes (being the characteristic of DASH diet i.e. sodium, potassium, magnesium, calcium and vitamin D) between the groups at the end of the study.

Our findings also revealed that reductions in weight and obesity indices were significantly greater in DASH group than CRD group, after adjusting for the confounders (Table 3). There is evidence indicating that as DASH diet includes high fruits, vegetables, dietary fiber and calcium as well as low fat intake, particularly in the form of dairy products, and also simple sugar, following DASH diet is an effective approach in weight loss in obesity and a number of metabolic diseases [31–33]. For example, Asemi et al. [54] showed that adherence to DASH diet compared with usual low-calorie diet in patients with PCOS for 8 weeks resulted in greater reductions in weight and BMI. Similar findings were also reported by subsequent studies conducted on patients with NAFLD [40, 55]. Meanwhile, Rifai et al. [56] in patients with heart failure failed to show any noticeable effect on weight and BMI after 3 months. In a randomized controlled trial in 2021, 12-week following DASH diet with and without exercise on anthropometric indices, DASH diet plus exercise resulted in significantly lower weight and WHR, although at the end of the study, the inter-group differences were not statistically significant [57]. Moreover, a cross-sectional study on 305 overweight and obese women showed that adherence to DASH diet was inversely associated with greater weight reduction [58]. Soltani et al. [42] in a meta-analysis of randomized controlled trials on the effect of low calorie DASH diet on weight (N = 10), BMI (N = 6) and WC (N = 2) demonstrated its lowering effect on the studied anthropometric measures. It appears that DASH diet because of its low energy density, high content of dietary fiber, particularly due to the higher intake of whole grains and vegetables could be effective in delaying carbohydrate absorption and increasing satiety [42].

Although the results of the present trial failed to show any difference in micronutrients such as calcium and magnesium between the two diets at the end of the study, cummulative evidence shows that DASH diet contains high calcium and magnesium. Studies have demonstrated that dietary calcium increases lipolysis and plays an important role in weight management [34, 59]. Hence, calcium and magnesium intakes are inversely related with obesity due to their roles in the saponificantion of fatty acids [34]. On the other hand, increased sodium intake-which is low in DASH diet-results in fat accumulation through increasing leptin [42]. Therefore, following DASH diet for long term appears to help in weight control.

Previous studies have also demonstrated that high sodium intake is associated with the risk of NAFLD [60]**.** Uetake et al. [61]. found that high-salt diet exacerbated nonalcoholic steatohepatitis in high-fat diet-fed lipoprotein receptor-1 (LOX-1) transgenic /apoE knockout mice and that this effect was associated with the induction of oxidative and inflammatory processes. Oxidative stress and chronic inflammation play a major role in pathophysiology of NAFLD [62]**.** A high-salt diet also activates the aldose reductase-fructokinase pathway in the liver and hypothalamus, which leads to endogenous fructose production with the development of leptin resistance and hyperphagia that cause obesity, IR, and NAFLD [63]**.**

Our results also failed to find any significant difference in serum FBS change after 8 weeks between the groups, however, a significant reduction in serum HbA1c was observed in DASH group (*P* < 0.001) compared with an increase in control group, after adjusting for the confounding factors (*p* < 0.001) (Table 3). There is evidence with a great emphasis on the consumption of whole grains, fruits and vegetables in DASH diet, therefore, the high content of dietary fiber in DASH diet decreases carbohydrate absorption and lowers blood glucose level [54]. Furthermore, DASH diet also includes food items with low glycemic index and low energy content which is efficient not only in hypertension but also could be considered as an efficient dietary approach in the management of IR-related chronic diseases [54, 64]. Shirani et al. [65] have reported that the adherence to DASH diet is more likely to be associated with lower risk of hyperglycemia.

In this study, apart from PLR (*p* = 0.019), there were no significant inter-group differences in WBC-derived inflammatory biomarkers at the end of study. Nevertheless, serum levels of TLR-4, MCP-1 and LPS decreased significantly in DASH group while no changes were found in control group. Even after adjusting for confounding variables, between-group analysis showed significant differences in serum concentrations of TLR-4, MCP-1 and LPS (Table 3). Fung et al. [66] in a prospective cohort study showed that DASH diet was associated with lower serum interleukin-6 (IL-6) and C- reactive protein (CRP). Holt et al. [67] also illustrated that high consumption of fruits and vegetables decreased IL-6, CRP and TNF-α. Similar findings have been reported on patients with metabolic syndrome [68], T2DM [69], Mets [70] and NAFLD [40]. DASH diet has shown favorable effects on serum CRP and hs-CRP levels been through previous studies [55, 71, 72]. However, Asemi et al. [73] on those with gestational diabetes (24–24 weeks) reported no effect of DASH diet on CRP level. Studies investigating the effect of DASH diet with other inflammatory biomarkers are few. Taheri et al. [58] showed that DASH diet compared with other dietary pattern did not affect serum levels of MCP-1. A systematic review on 16 observational and 13 interventional studies concluded that plant-based dietary patterns (such as Mediterranean or DASH diet) reduce inflammatory biomarkers such as serum hs-CRP, TNF-α and IL-6 [74].

Because DASH diet is rich in fruits and vegetables as well as flavonoids with antioxidant activity, adherence to DASH diet results in a decrease in free radicals, lipid peroxidation and inflammation and in turn, leads to reduced secretion of leptin and therefore, weight control [64]. Hence, considering the close link between oxidative stress and inflammation, inflammatory cells can produce large amounts of reactive oxygen species (ROS)- as a part of mechanism for immunological defense-to protect human organisms against invading pathogens [8].

Moreover, studies have demonstrated that the low glycemic index diet may decrease inflammation by slowing glucose absorption, altering gut microflora and therefore suppress the production of inflammatory cytokines, stimulate the production of short-chain fatty acids in intra-lumen which results in lower circulating FFA levels and thus subsequent inflammation [42]. Furthermore, parallel to the effect of DASH diet on meta-inflammation observed in serum levels of TLR-4 and MCP-1, the concentration of LPS decreased. Studies investigating the effect of DASH diet on serum LPS is limited. Observational and interventional studies have suggested that phytochemicals and other compounds present in DASH diet are directly or indirectly attributed in the modulation of inflammatory biomarkers as well as intestinal permeability and therefore, the body's susceptible to infection [75]. The link between obesity and high intestinal permeability has been well documented. The systemic levels of LPS are elevated in obese individuals through several mechanisms. e.g. impaired clearance in the liver, alterations in the gut microbiota, permeability, motility and enzyme levels, and serum levels of HDL-C [75]. Intercellular tight junctions can regulate intestinal permeability and factors such as fatty acids and proinflammatory cytokines are necessary to maintaining intestinal mucosa integrity [75]. Therefore, it appears obesity as well as impaired oxidative stress and chronic inflammation in response to the characteristics of DASH diet are involved in the improvement of inflammation.

Serum AST and ALT reduced significantly in DASH group whereas serum ALT decreased significantly in control group (Table 3). After adjusting for the confounders, there was only significant between-group difference in serum concentration of AST (p = 0.008) which is in line with other studies. For example, reduced levels of serum liver enzymes followed by DASH diet have been reported in patients with T2DM [69] and NAFLD [40, 76]. Moreover, Xiao et al. [55] in population-based cohort study illustrated that following DASH diet was less likely to be associated with NAFLD risk, particularly in women and those without abdominal obesity. Mahdavi et al. [71] also reported following DASH diet led to reductions in liver steatosis and fibrosis in male adolescents with hemophilia after 10 weeks. In the present study, the NNT was calculated for assessing the clinical importance of DASH diet. The estimated NNTs for 8-week following DASH diet compared with RCT for one and two grade improvements in NAFLD severity were found 2.5 and 6.67, respectively.

Our study had several strengths including studying NAFLD patients who newly diagnosed without receiving any medication or treatment, providing an individualized low-calorie diet as an approved strategy for NAFLD management) on the basis of DASH dietary pattern, good adherence to DASH diet by the patients and assessing specific inflammatory biomarkers compared with previous studies. However, lack of liver biopsy because of ethical considerations, not assessing other inflammatory factors as well as insulin resistance indices could be considered as the study limitations.

## Conclusion

It is concluded that following DASH diet for 8 weeks could significantly improve liver function in patients with NAFLD due to reduced weight and BMI, glycemic response, and meta-inflammation.

## Data Availability

The datasets used and/or analyzed during the current study are available from the corresponding author on a reasonable request.

## Abbreviations

ALT: Alanine aminotransferase
ANCOVA: Analysis of covariance
ARR: Absolute risk reduction
AST: Aspartate aminotransferase
BMI: Body mass index
BP: Blood pressure
CBC: Complete blood count
CI: Confidence interval
CRD: Calorie-restricted diet
CRP: C- reactive protein
DASH: Dietary Approaches to Stop Hypertension
DBP: Diastolic blood pressure
ELISA: Enzyme-linked immunosorbent assay
FBS: Fasting blood sugar
FFAs: Free fatty acids
HbA1c: Hemoglobin A1c
HC: Hip circumference
HDL-C: High-density lipoprotein cholesterol
HTN: Hypertension
IFCC: International Federation of Clinical Chemistry
IPAQ-SF: International physical activity questionnaire-short form
IR: Insulin resistance
LPS: Lipopolysaccharide
MAFLD: Metabolic associated fatty liver disease
MET: Metabolic equivalent of task
Mets: Metabolic syndrome
MCP-1: Monocyte chemoattractant protein
mHDL: Monocyte to high-density lipoprotein cholesterol ratio
MLR: Monocyte to lymphocyte ratio
MUFA: Monounsaturated fatty acids
NAFLD: Non-alcoholic fatty liver disease
NLR: Neutrophil to lymphocyte ratio
NNT: Number needed to treat
PCOS: Polycystic ovary syndrome
PLR: Platelets to to lymphocyte ratio
RAS: Random Allocation Software
RCT: Randomized clinical trial
SD: Standard deviation
SBP: Systolic blood pressure
SIRI: Systemic inflammation response index
T2DM: Type 2 diabetes mellitus
TLR-4: Toll-like receptors-4
TNF-α: Tumor necrosis factor-α
WBC: White blood cell
WC: Waist circumference
WHR: Waist to hip ratio
WHtR: Waist to height ratio
